# Sport Participation and Nutrition in Students: A Scoping Review of Neuroendocrine and Autonomic Mechanisms Linking Lifestyle Behaviors to Cognitive and Academic Outcomes

**DOI:** 10.3390/nu18111651

**Published:** 2026-05-22

**Authors:** Maria Giovanna Tafuri, Vincenzo Monda, Marco La Marra, Francesco Tafuri, Antonietta Messina, Antonietta Monda, Maria Casillo, Girolamo Di Maio, Domenico Tafuri, Francesca Latino, Fiorenzo Moscatelli, Rita Polito, Giovanni Messina

**Affiliations:** 1 Department of Literary, Linguistic and Philosophical Studies, Pegaso University, 80143 Naples, Italy; mariagiovanna.tafuri@unipegaso.it; 2 Department of Economics, Law, Cybersecurity, and Sports Sciences, University of Naples “Parthenope”, 80131 Naples, Italy; vincenzo.monda@uniparthenope.it; 3 Section of Human Physiology, Unit of Dietetics and Sports Medicine, Department of Experimental Medicine, University of Campania “Luigi Vanvitelli”, 80138 Naples, Italy; marco.lamarra@unicampania.it (M.L.M.); maria.casillo@unicampania.it (M.C.); 4 Department of Human Sciences, Link Campus University, 00166 Rome, Italy; francesco.tafuri@unilink.it; 5 Department of Precision Medicine, University of Campania “Luigi Vanvitelli”, 80138 Naples, Italy; antonietta.messina@unicampania.it; 6 Department of Human Science and Quality of Life Promotion, San Raffaele Telematic University, 00166 Rome, Italy; antonietta.monda@uniroma5.it; 7 Department of Psychology and Health Sciences, Pegaso Telematic University, 80143 Naples, Italy; girolamo.dimaio@unipegaso.it (G.D.M.); rita.polito@unipegaso.it (R.P.); 8 Department of Medical, Human Movement and Well-Being Sciences, University of Naples “Parthenope”, 80100 Naples, Italy; domenico.tafuri@uniparthenope.it; 9 Department of Biological and Environmental Sciences and Technologies, University of Salento, 73100 Lecce, Italy; 10 Department of Education and Sport Sciences, Pegaso Telematic University, 80143 Naples, Italy; 11 Department of Experimental Medicine, University of Campania “Luigi Vanvitelli”, 80138 Naples, Italy; giovanni.messina@unicampania.it

**Keywords:** physical activity, mediterranean diet, cognitive function, academic achievement, neuroendocrine mechanisms

## Abstract

**Background/Objectives:** Sport participation and nutrition are increasingly recognized as key determinants of cognitive function and academic achievement in student populations. However, the biological mechanisms underpinning these associations remain only partially understood. This scoping review aimed to map and synthesize the current evidence on neuroendocrine and autonomic mechanisms linking physical activity, sport participation, and nutrition to cognitive and academic outcomes in students. **Methods:** A systematic search of electronic databases was performed following PRISMA-ScR guidelines. Studies involving student populations that examined physical activity, sport participation, or dietary patterns in relation to cognitive function and/or academic performance were included. Particular attention was given to studies reporting biological or physiological indicators of underlying mechanisms, including neuroendocrine, autonomic, and brain-based measures. Data were extracted and synthesized qualitatively, with studies categorized according to the type of mechanistic evidence. **Results:** A total of 76 studies met the inclusion criteria. The available evidence was more extensive for physical activity, sport participation, and fitness-related exposures than for nutrition-related variables or integrated lifestyle models. Cognitive outcomes, particularly executive function, attention, working memory, and memory performance, were assessed more frequently and showed more consistent associations with lifestyle behaviors than academic outcomes, which were less commonly and more heterogeneously evaluated. Mechanistic evidence was unevenly distributed: only a limited subset of studies included direct biological or psychophysiological measures, mainly neuroimaging, brain-derived neurotrophic factors, cortisol-related indices, or heart rate variability. In contrast, inflammatory, metabolic, and gut microbiota-related mechanisms were mostly discussed at a conceptual or indirect level. Overall, the findings indicate a broad associative literature but a relatively small body of studies directly testing biological pathways linking physical activity, nutrition, cognition, and academic performance. **Conclusions:** Current evidence indicates potential associations between sport participation, nutrition, cognitive outcomes, and multiple biological pathways. However, the scoping nature of the review, the predominance of observational designs, and the limited use of direct mechanistic assessments prevent firm causal conclusions. Future research should prioritize longitudinal and intervention studies integrating behavioral, nutritional, cognitive, academic, and biological measures within the same design.

## 1. Introduction

Cognitive function and academic performance in students are influenced by multiple behavioral and physiological factors, including physical activity, nutrition, sleep, stress regulation, and metabolic health. Among these factors, sport participation and physical activity have received considerable attention because they are frequently associated with executive function, attention, memory, and school-related outcomes. Previous reviews have shown that physical activity and fitness are generally linked to cognitive benefits in children and adolescents, although evidence for academic achievement is more heterogeneous and depends strongly on study design, population characteristics, and outcome definition [1,2,3,4,5,6].

Physical activity and sport participation may influence cognition through several biological pathways, including neurotrophic signaling, autonomic regulation, stress-related hormonal responses, and brain functional adaptations [7,8,9,10,11]. However, many studies in student populations remain observational and do not directly measure these pathways. Nutrition represents a second lifestyle domain of potential relevance. Breakfast consumption, overall diet quality, Mediterranean dietary patterns, and nutrient availability have been associated with cognitive and educational outcomes, but the evidence is more heterogeneous and less frequently connected to direct biological measurements. Importantly, physical activity and nutrition are often studied separately, although they may converge on shared neuroendocrine, autonomic, inflammatory, metabolic, and gut–brain mechanisms [8,9,10,11,12,13,14,15,16,17].

This gap is especially relevant in student populations. In this review, the term “students” is used broadly to include individuals engaged in formal educational settings, encompassing primary school children, secondary school adolescents, and university students. These groups were considered together because they share exposure to structured learning environments and sustained cognitive demands; however, developmental differences across educational levels were considered during the synthesis, particularly when interpreting cognitive outcomes, academic indicators, and mechanistic evidence [12,18].

Given the breadth and heterogeneity of the available evidence, a scoping review was considered appropriate to map how the literature has investigated the relationships among lifestyle behaviors, cognitive function, academic outcomes, and biological pathways. The purpose of this review was not to estimate pooled effect sizes or establish causal mechanisms, but to clarify the extent, distribution, and nature of the available evidence and to identify where direct mechanistic data are still lacking. Scoping reviews are specifically designed to map complex and heterogeneous bodies of literature, identify knowledge gaps, and clarify how research in a given field has been conducted [19].

Accordingly, the objectives of this review were to: (i) map the available evidence linking sport participation, physical activity, and nutrition to cognitive and academic outcomes in student populations; (ii) describe how neuroendocrine, autonomic, inflammatory, metabolic, and gut microbiota-related pathways have been measured or conceptualized; (iii) distinguish studies providing direct biological evidence from those relying on physiological proxies or theoretical inference; (iv) identify methodological gaps limiting causal interpretation and translation into educational and public health settings [20,21].

## 2. Materials and Methods

### 2.1. Study Design

This study was conducted as a scoping review to map and synthesize the available evidence on the relationships among sport participation, nutrition, cognitive function, and academic achievement in students, with a specific focus on neuroendocrine, autonomic, inflammatory, metabolic, and microbiota-related mechanisms. A scoping review methodology was selected due to the conceptual breadth of the topic, the heterogeneity of exposures and outcomes, the expected variability in study designs, and the need to clarify how mechanistic pathways have been investigated across different student populations.

The review was designed and reported in accordance with the Preferred Reporting Items for Systematic Reviews and Meta-Analyses extension for Scoping Reviews (PRISMA-ScR) and followed the methodological guidance provided by the Joanna Briggs Institute (JBI) for scoping reviews [8].

### 2.2. Eligibility Criteria

Studies were considered eligible if they met the following criteria:

(i) Included students engaged in formal educational settings, including school-aged children, adolescents, or university students.
(ii) Examined at least one exposure related to sport participation, physical activity, exercise, physical fitness, nutritional behaviors, dietary patterns, or nutrition-related factors.
(iii) Reported outcomes related to cognitive function and/or academic achievement.
(iv) Investigated or explicitly addressed at least one biological or psychophysiological pathway relevant to neuroendocrine, autonomic, inflammatory, metabolic, or gut microbiota-related regulation. Mechanistic relevance was assigned only when the study either: (a) directly measured biological markers or neurophysiological variables; (b) assessed validated physiological proxies, such as heart rate variability or neuroimaging indices; (c) explicitly tested or discussed a predefined mechanistic hypothesis linking lifestyle exposure, cognition, or academic outcomes.
(v) Were original peer-reviewed articles.

Studies were excluded if they:

(i) Focused exclusively on clinical populations without a student or educational context, unless the sample was clearly composed of students;
(ii) Did not report cognitive or academic outcomes;
(iii) Addressed only general mental health or quality-of-life outcomes without relevance to cognition or academic performance;
(iv) Were editorials, letters, conference abstracts, narrative commentaries, dissertations, protocols, or reviews;
(v) Were not published in English.

### 2.3. Information Sources and Search Strategy

A systematic literature search was conducted in the following electronic databases: PubMed/MEDLINE, Scopus, Web of Science, Embase, PsycINFO, and SPORTDiscus. The search covered the period from database inception to February 2026.

To ensure completeness, the reference lists of all included studies and relevant review articles were manually screened to identify additional eligible records.

The search strategy combined controlled vocabulary terms (e.g., MeSH) and free-text keywords related to four main domains:

(1) Student populations (“student*”, “schoolchild*”, “adolescent*”, “college student*”, “university student*”);
(2) Exposures (“sport participation”, “physical activity”, “exercise”, “fitness”, “diet”, “nutrition”, “breakfast”, “Mediterranean diet”, “dietary pattern*”);
(3) Outcomes (“cognitive function”, “executive function”, “attention”, “working memory”, “learning”, “academic achievement”, “school performance”, “grade point average”, “GPA”, “standardized test*”);
(4) Mechanistic pathways (“neuroendocrine”, “autonomic”, “heart rate variability”, “HRV”, “cortisol”, “brain-derived neurotrophic factor”, “BDNF”, “inflammation”, “cytokines”, “microbiota”, “gut–brain axis”, “extracellular vesicles”, “Klotho”, “GPLD1”).

Boolean operators (AND, OR) were used to combine search terms, and truncation was applied where appropriate.

### 2.4. Study Selection

All records retrieved from the searches were exported into a reference management software program, and duplicates were removed. The remaining studies were then imported into a screening platform for eligibility assessment.

Two reviewers independently screened titles and abstracts against the predefined inclusion and exclusion criteria. Full texts were retrieved for all records deemed potentially eligible. The same two reviewers independently assessed the full texts for final inclusion. Discrepancies at either stage were resolved through discussion and, when necessary, by consultation with a third reviewer.

The study selection process was documented using a PRISMA-ScR flow diagram.

### 2.5. Data Charting Process

Data extraction was performed using a standardized charting form developed a priori and pilot-tested on a sample of included studies. Two reviewers independently extracted the data, and discrepancies were resolved by consensus.

The charting form was iteratively refined during the review process to ensure that all relevant information was captured consistently, reflecting the exploratory and flexible nature of scoping review methodology.

### 2.6. Data Items

For each included study, the following information was extracted:

- Bibliographic information (authors, year, country);
- Study design and temporal framework (e.g., cross-sectional, longitudinal, interventional);
- Population characteristics (age, sex, educational level, sample size);
- Exposure characteristics (type of sport participation, physical activity, exercise, fitness, dietary behavior, nutritional exposure, or dietary pattern);
- Comparator, when applicable;
- Cognitive outcomes (e.g., executive functions, attention, memory, processing speed, learning);
- Academic outcomes (e.g., grades, GPA, standardized tests, school performance indicators);
- Mechanistic variables, biomarkers, or pathways assessed (e.g., BDNF, cortisol, HRV, inflammatory markers, microbiota-related measures, exerkines, extracellular vesicles);
- Methodological approaches used to assess biological mechanisms, when available;
- Main findings relevant to the relationships among exposure, mechanism, and outcome;
- Key methodological characteristics or limitations reported by the authors.

In the main text, a focused mechanistic table was created to summarize only those studies that provided direct biological measures or clearly operationalized physiological proxies relevant to the review question. Studies in which mechanisms were only theoretically inferred were retained in the overall descriptive synthesis but were not included in the focused mechanistic table, in order to avoid conflating associative findings with direct mechanistic evidence.

### 2.7. Classification of Evidence

To enhance conceptual clarity, included studies were grouped into the following categories:

(i) Studies focusing primarily on sport participation, physical activity, exercise, or fitness;
(ii) Studies focusing primarily on nutrition or dietary exposures;
(iii) Studies examining both physical activity-related and nutrition-related exposures;
(iv) Studies assessing cognitive outcomes only;
(v) Studies assessing academic outcomes only;
(vi) Studies explicitly examining biological mediators or mechanistic pathways.

Because only a subset of the included studies explicitly assessed biological mediators, mechanistically informative studies were synthesized separately in a dedicated table in the Section 3. This selective approach was adopted to preserve the specificity of mechanistic interpretation and to avoid conflating associative evidence with studies providing direct biological measurement. As a result, the number of studies included in the mechanistic summary table is intentionally limited.

Mechanistic variables were further classified into four broad domains:

(1) Neuroendocrine pathways;
(2) Autonomic pathways;
(3) Inflammatory and metabolic pathways;
(4) Gut microbiota-related pathways.

Additionally, mechanistic evidence was qualitatively categorized into three levels: direct biological evidence was defined as evidence derived from objective biological, biochemical, neuroimaging, or neurophysiological measurements, such as BDNF, cortisol, inflammatory markers, microbiota-related markers, fMRI, or structural neuroimaging. Indirect physiological evidence was defined as evidence based on validated physiological proxies plausibly reflecting biological regulation, such as heart rate variability or other autonomic indices. Inferred mechanistic evidence was defined as evidence in which a biological pathway was proposed or discussed without direct measurement of the mechanism within the study design. This classification was used to improve transparency and to limit overinterpretation of mechanistic claims.

### 2.8. Critical Appraisal

Although a formal risk-of-bias assessment was not used as an exclusion criterion, key methodological features were systematically recorded to support interpretation of the evidence. These included study design, sample size, population characteristics, exposure assessment, cognitive and academic outcome measures, presence or absence of biological measurements, and whether the study design allowed any temporal or mechanistic inference. These elements were used to contextualize the strength of evidence across domains without producing pooled quality scores.

### 2.9. Synthesis of Results

Given the expected heterogeneity in study populations, exposures, outcomes, and mechanistic measures, results were synthesized using a descriptive and thematic approach rather than through quantitative meta-analysis [8].

The synthesis was structured to reflect the main objectives of the review:

(1) Mapping the evidence linking sport participation and nutrition to cognitive and academic outcomes in students;
(2) Identifying which neuroendocrine, autonomic, inflammatory, metabolic, and microbiota-related pathways have been investigated;
(3) Examining how these mechanistic pathways have been conceptualized and operationalized across studies;
(4) Highlighting key evidence gaps and methodological challenges.

Where appropriate, findings were summarized in tables according to study design, population characteristics, exposure type, outcome category, and biological mechanisms investigated.

## 3. Results

**Figure 1:**
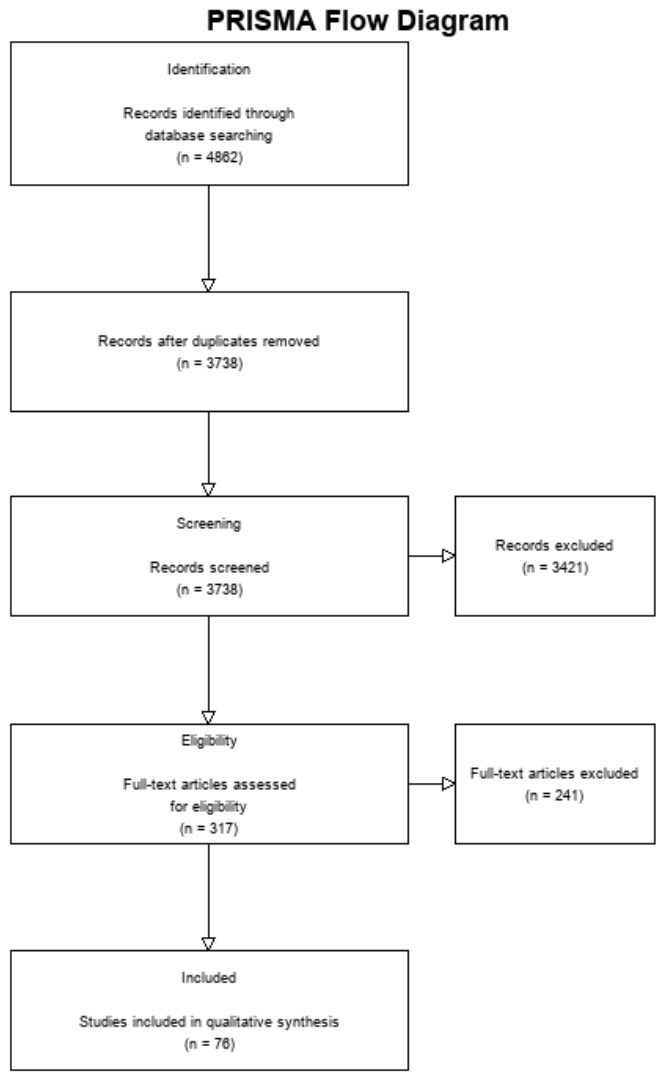
PRISMA flow diagram of the study selection process. The diagram illustrates the identification, screening, eligibility, and inclusion of studies in this scoping review. A total of 4862 records were initially identified through database searching, of which 3738 remained after duplicate removal. Following title and abstract screening, 3421 records were excluded. A total of 317 full-text articles were assessed for eligibility, with 241 studies excluded based on predefined criteria. Ultimately, 76 studies were included in the qualitative synthesis [8].

The database search yielded a total of 4862 records. After removal of duplicates (n = 1124), 3738 unique records remained and were screened by title and abstract. Of these, 3421 records were excluded because they did not meet the predefined inclusion criteria. A total of 317 full-text articles were assessed for eligibility. Following full-text review, 241 studies were excluded for the following reasons: absence of relevant cognitive or academic outcomes (n = 78), lack of student populations (n = 52), absence of mechanistic relevance (n = 63), and inappropriate study design (n = 48). Ultimately, 76 studies met the inclusion criteria and were included in the qualitative synthesis. The study selection process is summarized in the PRISMA-ScR flow diagram (Figure 1) [21].

As shown in Table 1, the evidence base was broad but unevenly distributed across populations, exposure domains, outcomes, and mechanistic levels. Physical activity, sport participation, exercise, and fitness-related exposures were more extensively represented than nutrition-related variables, whereas integrated physical activity–nutrition models were comparatively limited. Cognitive outcomes were assessed more frequently than academic outcomes, and direct biological or psychophysiological evidence was available only in a restricted subset of studies. This descriptive mapping supports a cautious interpretation of mechanistic findings and justifies the focused synthesis of studies providing direct or indirect mechanistic evidence.

### 3.1. Characteristics of Included Studies

The included studies (n = 76) encompassed primary school children, adolescents, and university students. This broad population framework was retained because the review aimed to map evidence across formal educational settings; however, findings were interpreted with attention to developmental stage. Studies involving school-aged children more frequently examined executive function, attention, and school performance, whereas studies involving adolescents and university students more often addressed lifestyle behaviors, stress-related mechanisms, diet quality, or academic indicators such as grades and GPA. Overall, the evidence base was heterogeneous in terms of age group, study design, exposure assessment, and outcome definition.

The studies presented in Table 2 were selected because they provided the clearest examples of direct biological or validated physiological mechanistic assessment within student populations. The table is therefore illustrative rather than exhaustive and is intended to support the focused mechanistic interpretation of the broader evidence base.

Participants included school-aged children, adolescents, and university students, with sample sizes ranging from small experimental cohorts (n < 30) to large observational studies involving several thousand participants. Most studies were conducted in educational settings across Europe, North America, and Asia, with a smaller number of studies originating from other regions.

In terms of study design, the evidence base was predominantly composed of cross-sectional and observational studies, although a number of longitudinal and intervention studies were also identified. Experimental studies were more frequently observed in research focusing on mechanistic outcomes, particularly neuroendocrine and physiological markers.

Regarding exposures, the included studies were categorized into three main groups: those focusing on sport participation or physical activity, those examining nutritional or dietary factors, and those investigating combined lifestyle behaviors. Physical activity-related exposures varied widely in terms of frequency, intensity, and modality, while nutritional exposures ranged from single behaviors (e.g., breakfast consumption) to overall dietary patterns such as adherence to the Mediterranean diet.

Cognitive outcomes were assessed using a variety of standardized neuropsychological tests, with particular emphasis on executive functions, attention, working memory, and processing speed. Academic outcomes were typically measured using grade point average, school grades, or standardized test scores.

A subset of studies explicitly investigated biological mechanisms, including neuroendocrine markers (e.g., BDNF, cortisol), autonomic indicators (e.g., heart rate variability), inflammatory and metabolic markers, and gut microbiota-related variables. However, the methodological approaches used to assess these mechanisms varied substantially across studies, contributing to heterogeneity in the evidence base. This heterogeneity was particularly evident in the assessment of biological mechanisms, which were often inconsistently operationalized across studies. In many cases, mechanistic pathways were inferred rather than directly measured, further limiting the number of studies that could be included in the focused mechanistic synthesis.

Overall, the included studies demonstrated considerable variability in design, measurement approaches, and conceptual frameworks, supporting the need for a structured mapping and synthesis of the available evidence.

Across the broader evidence base, mechanistic assessment was inconsistently operationalized; therefore, studies with direct autonomic, neuroendocrine, inflammatory/metabolic, or microbiota-related measures were examined separately to clarify how biological mediation has been addressed in the literature [27].

### 3.2. Distribution of Evidence Across Exposure Domains

The distribution of evidence across exposure domains was clearly uneven. Physical activity, sport participation, exercise, and fitness-related variables represented the largest body of evidence, including studies on habitual physical activity, organized sport, acute exercise, school-based movement programs, and cardiorespiratory fitness. Nutrition-related studies were fewer and more heterogeneous, focusing mainly on breakfast consumption, diet quality, dietary patterns, Mediterranean diet adherence, or nutrition education. Only a small proportion of studies examined physical activity and nutrition within the same analytical framework. This imbalance is important because it indicates that most mechanistic hypotheses in the current literature are anchored to physical activity-related pathways, whereas nutrition-related mechanisms remain less frequently and less directly tested in student populations.

### 3.3. Cognitive and Academic Outcome Patterns

With regard to outcomes, a clear pattern emerged across the included studies, with cognitive measures being more consistently and extensively assessed than academic indicators. Most studies focused on domains such as executive functions, attention, working memory, and processing speed, reflecting a strong interest in the more proximal and measurable aspects of cognitive functioning. These outcomes were typically evaluated through standardized neuropsychological tests or computerized tasks, allowing for relatively precise and comparable assessments across different study designs.

In contrast, academic outcomes were less frequently investigated and, when included, were often operationalized in a more heterogeneous manner. Common indicators included grade point average, school grades, standardized test scores, or general measures of academic performance. However, these variables are inherently influenced by a broader set of contextual factors, including socioeconomic background, educational environment, and individual motivation, which may partly explain their less consistent use in the literature.

Another relevant aspect concerns the relationship between cognitive and academic outcomes. While several studies implicitly assume that improvements in cognitive function may translate into better academic performance, relatively few directly tested this link within the same design. As a result, the connection between cognitive gains and educational outcomes often remains inferred rather than empirically demonstrated.

Importantly, the relationship between cognitive improvement and academic achievement was not consistently tested. In several studies, cognitive outcomes were interpreted as educationally relevant because executive function, attention, and memory are theoretically linked to learning. However, relatively few studies simultaneously assessed cognitive performance, academic achievement, and biological mechanisms within the same design. Therefore, the pathway from lifestyle exposure to biological regulation, cognitive change, and real academic performance remains only partially supported by direct empirical evidence.

### 3.4. Mechanistic Evidence: Overview

Mechanistic evidence varied substantially across studies. Direct biological evidence was mainly available for physical activity-related exposures and included neuroimaging measures, BDNF assessment, and autonomic indicators such as HRV. Indirect physiological evidence was also more frequently linked to physical activity than to nutrition. In contrast, nutrition-related mechanisms were commonly inferred from known biological pathways rather than directly measured in student samples. Inflammatory, metabolic, and gut microbiota-related pathways were the least directly investigated domains. These findings indicate that the mechanistic literature is still at an early stage and that most available studies do not simultaneously assess lifestyle exposure, biological mediator, cognitive outcome, and academic performance [18]. However, part of the mechanistic rationale derives from broader translational and adult-based literature rather than from student-specific investigations, further highlighting the limited direct mechanistic evidence currently available in educational populations.

Across the included literature, mechanistic evidence was highly heterogeneous in both conceptualization and measurement. Some studies incorporated objective biological markers, such as neuroendocrine indicators or autonomic measures, while others relied on indirect proxies or theoretical interpretations of potential pathways. As a result, the level of mechanistic depth varied substantially, ranging from studies providing clearly operationalized biological data to those in which mechanisms were only discussed in the interpretation of results. Among the studies that did include direct or semi-direct measures, autonomic and neuroendocrine processes appeared to be the most frequently explored domains. Indicators such as heart rate variability, stress-related hormonal responses, and psychophysiological regulation were more commonly assessed, particularly in relation to physical activity and exercise. In contrast, other pathways—such as inflammatory processes, metabolic regulation, or gut–brain interactions—were less consistently represented and often examined in more indirect ways. This uneven distribution of mechanistic evidence reflects, at least in part, the methodological challenges associated with integrating biological measurements into studies conducted in educational or real-world settings. It also highlights a broader limitation of the field: while there is growing recognition of the importance of biological mediation, relatively few studies adopt designs that simultaneously capture exposure, mechanism, and cognitive or academic outcomes within a unified framework. For this reason, the focused mechanistic synthesis presented in Table 2 was intentionally restricted to studies providing the most explicit and directly assessed biological information. This approach allows for a clearer interpretation of the available evidence, while also making evident the current gaps and the need for more integrative and methodologically robust research in this area.

### 3.5. Neuroendocrine Pathways

Neuroendocrine mechanisms represent one of the most frequently discussed pathways linking sport participation and, to a lesser extent, nutrition with cognitive function in student populations. Several studies have suggested that physical activity may influence brain-related processes through the modulation of neurotrophic factors, stress-related hormones, and other signaling molecules involved in neural plasticity and cognitive regulation [28].

For physical activity and sport participation, neuroendocrine evidence was mainly related to neurotrophic signaling and stress regulation. Studies assessing BDNF or brain-based adaptations suggest that exercise may be associated with biological processes relevant to learning and memory, although most findings remain limited by small samples, short interventions, or cross-sectional designs.

Exercise has been consistently associated with the upregulation of neurotrophic factors such as brain-derived neurotrophic factor (BDNF), which is widely recognized for its role in synaptic plasticity, learning, and memory processes. Although direct measurement of BDNF or similar markers was not systematically available across all studies, the underlying rationale was often supported by a growing body of experimental and translational research. In student-based settings, however, the incorporation of these biomarkers remains relatively limited, and in many cases their role is inferred rather than directly assessed [29].

Alongside neurotrophic signaling, stress-related hormonal responses—particularly those involving cortisol—were also considered relevant. Regular engagement in physical activity has been linked to improved regulation of the hypothalamic–pituitary–adrenal (HPA) axis, potentially contributing to more adaptive stress responses and, consequently, to better cognitive functioning. Some studies further suggested that more balanced neuroendocrine profiles may support attentional control and executive functioning, especially in contexts characterized by academic demands.

Compared to physical activity, the role of nutrition in modulating neuroendocrine pathways was less consistently addressed. While certain dietary patterns and nutrient profiles have been hypothesized to influence neurotrophic and hormonal regulation, the available evidence within student populations remains fragmented and often indirect. As a result, nutrition-related neuroendocrine mechanisms are less clearly defined and require further empirical investigation [30].

For nutrition, the neuroendocrine pathway was less directly documented. Dietary patterns may plausibly influence neurotrophic signaling, glucose availability, hormonal regulation, and stress responsiveness, but few studies in student populations directly measured these mechanisms. Therefore, nutrition-related neuroendocrine interpretations should be considered hypothesis-generating rather than conclusive.

Overall, the findings suggest that neuroendocrine processes constitute a plausible and increasingly supported pathway through which lifestyle behaviors may influence cognitive outcomes. However, the limited number of studies directly measuring these mechanisms in student populations indicates that current evidence is still emerging and would benefit from more integrative designs combining behavioral, biological, and cognitive assessments.

### 3.6. Autonomic Pathways

Autonomic regulation represents another key pathway through which sport participation, and, to a lesser extent, nutritional behaviors may influence cognitive function in student populations. Compared to neuroendocrine markers, autonomic indicators—particularly heart rate variability (HRV)—have been more frequently and directly assessed in applied and educational settings, making them one of the most tangible links between lifestyle factors and cognitive outcomes [31].

Across the included studies, higher levels of physical activity and better fitness profiles were generally associated with more adaptive autonomic patterns, typically reflected in increased HRV and improved balance between sympathetic and parasympathetic activity. These physiological adaptations are often interpreted as markers of greater flexibility in stress regulation and enhanced capacity to respond to environmental and cognitive demands. In this context, autonomic regulation has been linked to key cognitive domains such as attention, executive functioning, and processing speed.

Some studies also explored the relationship between autonomic measures and task-related performance, suggesting that individuals with more favorable autonomic profiles may exhibit more efficient cognitive processing and better behavioral responses during cognitively demanding tasks. This perspective aligns with the broader view of HRV as an index of central–autonomic integration, reflecting the functional interplay between brain networks and peripheral physiological systems.

In contrast, the role of nutrition in shaping autonomic function was less consistently addressed within the included literature. While certain dietary patterns have been associated with autonomic regulation in broader populations, evidence in student-based samples remains limited and often indirect. As a result, the contribution of nutrition to autonomic pathways in relation to cognition and academic performance is still insufficiently characterized [32].

Overall, autonomic mechanisms appear to provide one of the most accessible and empirically supported pathways linking physical activity to cognitive function in student populations. At the same time, the relatively limited integration of autonomic measures with academic outcomes and nutritional variables highlights an important direction for future research, particularly in studies aiming to adopt a more comprehensive and multidimensional approach.

Overall, autonomic evidence was stronger for physical activity than for nutrition. HRV and related autonomic indices provide a measurable link between lifestyle behaviors and cognitive regulation, but studies combining autonomic assessment with academic outcomes remain scarce. This limits the possibility of determining whether autonomic adaptations translate into measurable educational benefits.

### 3.7. Inflammatory and Metabolic Pathways

Inflammatory and metabolic processes represent another potential pathway through which lifestyle behaviors may influence cognitive function and academic performance. However, compared to neuroendocrine and autonomic mechanisms, these pathways were less consistently and less directly investigated within the included studies.

A limited number of studies suggested that regular physical activity may contribute to a more favorable inflammatory profile, potentially through the reduction in low-grade systemic inflammation and improvements in metabolic regulation. These adaptations have been proposed as relevant for brain health, given the growing evidence linking chronic inflammation and metabolic dysregulation to impairments in cognitive function. In student populations, however, direct assessments of inflammatory markers—such as C-reactive protein or other cytokines—were relatively rare, and their relationship with cognitive or academic outcomes was often inferred rather than explicitly tested [33].

Similarly, metabolic factors such as glucose regulation, lipid profiles, and body composition were occasionally considered, particularly in studies focusing on broader health-related outcomes. While these variables may plausibly influence cognitive processes through energy availability and neural efficiency, their role as mediators was seldom examined in a structured or integrated manner within the same analytical framework.

Nutrition-related studies offered some additional insights into these pathways, particularly in relation to dietary quality and metabolic health. Nonetheless, even in this domain, the connection between metabolic or inflammatory status and cognitive or academic outcomes was often indirect, with few studies simultaneously assessing dietary exposures, biological markers, and cognitive performance.

Overall, inflammatory and metabolic mechanisms should be interpreted cautiously in the context of this review. Although these pathways are biologically plausible and supported by broader literature on brain health and lifestyle behaviors, they were rarely measured directly in the included student-based studies. Consequently, their role in linking physical activity or nutrition to cognitive and academic outcomes remains largely inferential.

### 3.8. Gut Microbiota-Related Pathways

Gut microbiota-related mechanisms have recently emerged as a potentially important pathway linking nutrition, and to a lesser extent physical activity, with cognitive function. However, within the context of the present review, this domain was only marginally represented and remains one of the least explored areas in student populations.

The theoretical basis for this pathway is grounded in the concept of the gut–brain axis, a bidirectional communication system involving neural, endocrine, immune, and metabolic signaling. Dietary patterns, nutrient intake, and overall eating behaviors are known to influence the composition and diversity of the gut microbiota, which in turn may affect brain function through multiple biological routes. These include modulation of neurotransmitter production, regulation of inflammatory processes, and interactions with stress-related pathways [34].

Despite this growing body of knowledge, very few of the included studies directly assessed microbiota-related variables. In most cases, the role of the gut–brain axis was discussed at a conceptual level, often in relation to dietary quality or specific nutritional habits, rather than being empirically measured through microbiome analyses or related biomarkers. This limits the possibility of drawing clear conclusions about the contribution of microbiota-mediated mechanisms in student populations [35].

Physical activity has also been suggested to influence gut microbiota composition, potentially interacting with dietary factors to shape metabolic and neurobiological outcomes. However, this interaction remains largely unexplored within the educational and student-focused literature, where integrated assessments of physical activity, nutrition, microbiota, and cognitive outcomes are still rare.

Overall, gut microbiota-related pathways remain a preliminary and largely theoretical area within the student-focused literature included in this review. Although the gut–brain axis provides a plausible framework linking diet, metabolism, immune regulation, and cognition, direct microbiota assessments were rarely incorporated. Therefore, this domain should be presented as an emerging research gap rather than as an established mechanism in student populations.

### 3.9. Cross-Cutting Methodological Gaps

Across the included studies, several methodological limitations emerged consistently, highlighting important challenges in advancing a mechanistic understanding of the relationship between sport participation, nutrition, and cognitive or academic outcomes in student populations.

One of the most evident issues concerns the limited integration of behavioral, biological, and cognitive dimensions within the same study design. While many studies examined associations between lifestyle factors and cognitive outcomes, only a small proportion simultaneously incorporated objective biological measures, and even fewer directly tested these variables as mediators within a unified analytical framework. As a result, mechanistic interpretations are often based on indirect evidence rather than on clearly demonstrated causal pathways [36].

A second recurring limitation relates to the heterogeneity in both measurement approaches and study designs. Cognitive outcomes, academic indicators, and biological variables were operationalized using a wide range of tools and protocols, making direct comparisons across studies difficult. This variability is particularly pronounced in the assessment of biological mechanisms, where differences in measurement timing, type of biomarker, and analytical approach contribute to a fragmented evidence base.

Furthermore, a large proportion of the included studies adopted cross-sectional or observational designs, which, while valuable for identifying associations, do not allow for a robust evaluation of causal relationships. Intervention studies were present but often limited in duration, sample size, or scope, and only rarely incorporated multidimensional assessments capable of capturing changes across behavioral, biological, and cognitive domains simultaneously.

Another relevant gap concerns the underrepresentation of integrated lifestyle models. Physical activity and nutrition were frequently investigated as separate exposures, despite their likely interaction at both behavioral and biological levels. This separation may contribute to an incomplete understanding of how multiple modifiable factors jointly influence cognitive function and academic performance.

A further limitation concerns developmental heterogeneity. Primary school children, adolescents, and university students differ in neurodevelopmental stage, academic demands, autonomy over lifestyle behaviors, and exposure to environmental confounders. Pooling these groups conceptually is useful for mapping the field, but it also limits the specificity of mechanistic interpretation. Future studies should therefore examine whether biological pathways differ across educational levels and developmental stages. Another important gap concerns causal modeling. Most studies did not test mediation pathways linking lifestyle exposure, biological markers, cognitive function, and academic achievement. As a result, many proposed mechanisms remain biologically plausible but empirically unverified. Longitudinal and intervention studies incorporating repeated biological and cognitive assessments are needed to determine whether changes in neuroendocrine, autonomic, inflammatory, metabolic, or microbiota-related markers actually mediate changes in cognitive or academic outcomes.

Overall, these methodological limitations suggest that, although the field has made important progress in documenting associations, a more integrative and longitudinal approach is needed. Future research would benefit from study designs that combine precise exposure assessment, direct measurement of biological mechanisms, and robust evaluation of cognitive and academic outcomes within the same framework in order to move from associative evidence toward a more comprehensive understanding of underlying processes.

## 4. Discussion

This scoping review mapped the available evidence linking sport participation, physical activity, and nutrition to cognitive and academic outcomes in student populations, with particular attention to neuroendocrine, autonomic, inflammatory, metabolic, and gut microbiota-related pathways. The main finding is that the literature is broad but uneven. Physical activity and sport participation are more extensively studied than nutrition, cognitive outcomes are more frequently assessed than academic outcomes, and direct biological mechanisms are investigated in only a limited subset of studies.

The strongest and most coherent evidence concerns physical activity-related exposures and cognitive function. Across different study designs, physical activity, fitness, and structured exercise were frequently associated with executive function, attention, working memory, and memory performance. These findings are consistent with the broader literature suggesting that movement behaviors may support cognitive functioning through neuroplastic, neuroendocrine, and autonomic adaptations. However, even in this domain, the strength of inference varies considerably. Cross-sectional studies cannot establish directionality, and short-term interventions often do not clarify whether cognitive changes are sustained or translate into meaningful academic benefits.

Nutrition-related evidence was less developed and more heterogeneous. Studies addressing breakfast consumption, diet quality, Mediterranean diet adherence, or nutrition education suggest potential links with cognitive and educational outcomes, but these associations were less consistently connected to direct biological measures. This imbalance does not imply that nutrition is less relevant; rather, it indicates that nutrition-related pathways have been less frequently operationalized in mechanistic studies involving students. Future research should therefore give greater attention to dietary patterns, nutrient availability, metabolic regulation, and gut–brain interactions within designs that also include cognitive and academic endpoints.

The relationship between cognitive function and academic achievement remains one of the most important unresolved issues. Many studies assume that improvements in executive function, attention, or memory are educationally meaningful, but fewer directly demonstrate that these cognitive changes translate into grades, GPA, standardized test performance, or other real-world academic indicators. Academic outcomes are influenced by multiple contextual variables, including socioeconomic background, teaching quality, motivation, sleep, mental health, and family environment. Therefore, the link between lifestyle behaviors, cognition, and academic performance should be interpreted cautiously unless all three components are measured within the same study design [37].

From a mechanistic perspective, neuroendocrine and autonomic pathways appear to be the most empirically represented domains. Evidence involving BDNF, cortisol-related regulation, HRV, and neuroimaging measures provides plausible biological links between physical activity and cognitive function. Nevertheless, these mechanisms are not uniformly measured across studies and are rarely tested as mediators. Inflammatory, metabolic, and gut microbiota-related pathways are biologically plausible but remain comparatively underexplored in student populations. In particular, the gut–brain axis is often discussed as a theoretical framework rather than directly assessed through microbiome or related biomarker analyses.

Overall, the current literature should be interpreted as a mapping of potential pathways rather than a definitive demonstration of causal mechanisms. The predominance of observational studies, the heterogeneity of exposures and outcomes, and the limited integration of biological measures constrain the strength of mechanistic conclusions. The field would benefit from longitudinal and intervention studies that simultaneously assess physical activity, nutrition, biological mediators, cognitive performance, and academic outcomes. Such designs are necessary to move beyond associative models and to clarify whether neuroendocrine, autonomic, inflammatory, metabolic, or microbiota-related changes mediate the effects of lifestyle behaviors on educationally relevant outcomes [38].

## 5. Practical Implications

The findings of this review have several practical implications for educational, clinical, and policy contexts.

First, the frequent association between sport participation and cognitive function supports the educational relevance of structured physical activity within school and university settings. However, given the predominance of observational evidence, these findings should be translated into practice with appropriate caution and should not be interpreted as definitive proof of causal academic benefit.

From a nutritional perspective, dietary behaviors should be considered a relevant but still under-investigated component of student cognitive health. Regular eating patterns, breakfast consumption, adequate nutrient intake, and overall diet quality may contribute to cognitive functioning, but the mechanisms linking nutrition to academic outcomes remain less clearly defined than those related to physical activity. These aspects are particularly relevant in school and university contexts, where lifestyle habits are often shaped and consolidated.

Importantly, the results also highlight the need for a more integrated approach to lifestyle interventions. Physical activity and nutrition are frequently addressed separately in both research and practice, yet they likely interact through shared biological pathways. Educational programs and public health initiatives would benefit from adopting combined strategies that simultaneously target movement behaviors and dietary habits, rather than treating them as independent domains [39].

Another relevant implication concerns the role of biological monitoring in applied settings. While the use of biomarkers such as heart rate variability or stress-related indicators is still limited, their inclusion could offer valuable insights into individual responses to lifestyle interventions. This may support the development of more personalized and adaptive strategies aimed at optimizing both cognitive function and academic outcomes.

Overall, these findings suggest that promoting healthy lifestyle behaviors in student populations should be considered a multidimensional objective, with potential benefits extending beyond physical health to include cognitive and educational domains. However, translating these insights into practice requires further evidence from studies adopting integrated and mechanistically informed designs.

## 6. Limitations

This review should be interpreted considering several limitations, both inherent to the included literature and related to the methodological approach adopted. First, the overall heterogeneity of the studies represents a significant challenge. Differences in study design, population characteristics, exposure definitions, and outcome measures limited the possibility of direct comparison across studies and may have influenced the consistency of the observed patterns.

A specific limitation is related to the scope of the review question. Because this review aimed to map a broad and heterogeneous field, it included studies differing substantially in population, exposure, outcome, and mechanistic assessment. This breadth is appropriate for a scoping review but limits the possibility of making strong claims regarding effect magnitude, causality, or biological mediation.

A second limitation concerns the relatively limited number of studies providing direct biological measurements. Although the review specifically aimed to explore neuroendocrine, autonomic, inflammatory, metabolic, and microbiota-related mechanisms, most of the included studies did not directly assess these pathways. As a result, part of the mechanistic interpretation is necessarily based on indirect evidence or theoretical considerations rather than on consistently measured biological data.

In addition, the predominance of cross-sectional and observational designs restricts the ability to draw causal inferences. While these studies are valuable for identifying associations, they do not allow for a clear understanding of the directionality or underlying mechanisms of the observed relationships. Intervention studies were included but were often limited in scope, duration, or methodological integration [1].

Another aspect to consider is the potential influence of contextual and confounding variables, particularly in relation to academic outcomes. Factors such as socioeconomic status, educational environment, and individual differences in motivation or learning strategies may play a significant role but were not consistently accounted for across studies.

Finally, as a scoping review, the aim of this work was to map and synthesize the available evidence rather than to provide a quantitative estimate of effects. Consequently, no formal risk-of-bias assessment or meta-analysis was conducted. However, major methodological features, including study design, type of exposure assessment, outcome measurement, and presence or absence of biological markers, were considered when interpreting the strength and limitations of the evidence.

## 7. Conclusions

This scoping review shows that sport participation, physical activity, and nutrition are frequently linked to cognitive outcomes in student populations, whereas evidence regarding academic achievement is less direct and more heterogeneous. Neuroendocrine and autonomic pathways are the most represented mechanistic domains, particularly in studies focused on physical activity. In contrast, nutrition-related, inflammatory, metabolic, and gut microbiota-related mechanisms remain less frequently assessed through direct biological measures [4]. Overall, the field has advanced in documenting associations between lifestyle behaviors and cognition but remains limited in demonstrating causal biological pathways or direct translation to academic performance. Future research should adopt longitudinal and intervention designs integrating lifestyle exposures, biological mediators, cognitive measures, and academic outcomes within the same analytical framework.

## Figures and Tables

**Table 1:** Descriptive mapping of the included evidence according to population, exposure domain, outcomes, and mechanistic evidence.

Domain	Main Categories Identified	Overall Distribution in the Included Literature
Population	Primary school children; adolescents; university students	Evidence was distributed across different educational levels, with school-aged children and adolescents being frequently represented. University students were also included, particularly in studies addressing lifestyle behaviors, stress-related factors, and diet quality.
Study design	Cross-sectional, observational, longitudinal, experimental, interventional	The literature was predominantly composed of cross-sectional and observational studies. Longitudinal and interventional designs were less frequent but provided more informative evidence regarding temporal and mechanistic interpretation.
Exposure domain	Physical activity, sport participation, exercise, physical fitness	Physical activity-related exposures represented the most extensively investigated domain. Studies included habitual physical activity, organized sport participation, acute exercise, structured exercise interventions, school-based movement programs, and cardiorespiratory fitness.
Exposure domain	Nutrition, dietary behaviors, dietary patterns	Nutrition-related evidence was comparatively less developed and more heterogeneous. Studies mainly addressed breakfast consumption, diet quality, dietary patterns, Mediterranean diet adherence, nutrition education, and nutrient availability.
Integrated lifestyle exposure	Combined physical activity and nutrition	Studies examining physical activity and nutrition within the same analytical framework were limited, highlighting a relevant gap in the literature.
Cognitive outcomes	Executive function, attention, working memory, memory, processing speed, cognitive control	Cognitive outcomes were more frequently assessed than academic indicators. Executive function, attention, working memory, and memory were the most commonly examined domains.
Academic outcomes	Grades, grade point average, standardized test performance, school performance indicators	Academic outcomes were less consistently assessed and were operationalized using heterogeneous indicators. The direct link between cognitive improvements and real academic achievement was not consistently tested.
Direct biological evidence	Biomarkers, neuroimaging, neurophysiological measures	Direct biological evidence was available only in a limited subset of studies. These studies mainly involved BDNF, cortisol-related indices, neuroimaging measures, or other objective biological assessments.
Indirect physiological evidence	Heart rate variability, autonomic indices, psychophysiological measures	Indirect physiological proxies were more frequently linked to physical activity than to nutrition. HRV and related autonomic indicators represented one of the most accessible mechanistic measures.
Inferred mechanistic evidence	Conceptual discussion of neuroendocrine, autonomic, inflammatory, metabolic, and gut–brain pathways	Many studies discussed biological mechanisms conceptually without directly measuring them. This was particularly evident for nutrition-related, inflammatory, metabolic, and gut microbiota-related pathways.
Mechanistic domains	Neuroendocrine and autonomic pathways	Neuroendocrine and autonomic mechanisms were the most consistently represented mechanistic domains, especially in studies focused on physical activity and sport participation.
Mechanistic domains	Inflammatory, metabolic, and gut microbiota-related pathways	These mechanisms were less frequently assessed through direct measures and should be interpreted as emerging or hypothesis-generating domains within student populations.

**Table 2:** Selected studies providing direct or indirect mechanistic evidence linking lifestyle behaviors to cognitive or academic outcomes in student populations.

Study	Student Population	Exposure Domain	Study Design	Cognitive Outcomes	Academic Outcomes	Mechanistic Domain	Mechanistic Assessment	Main Mechanistic Contribution	Key Limitation	Level of Mechanistic Evidence
Chaddock-Heyman et al. (2014) [22]	School-aged children	Aerobic fitness/physical activity	Cross-sectional (neuroimaging study)	Executive function/cognitive control	Not directly assessed	Neuroendocrine/brain-based	fMRI	Direct evidence linking fitness with brain activation patterns underlying executive control	Cross-sectional design; no academic outcome	Direct
Hansen et al. (2003) [23]	Young adults (student population)	Autonomic regulation (resting vagal tone)	Observational	Working memory/attention	Not directly assessed	Autonomic	Heart rate variability (HRV)	Demonstrates association between vagal tone and cognitive performance	No direct sport exposure; indirect inference	Indirect
Hillman et al. (2009) [24]	School-aged children	Acute physical activity intervention	Experimental	Executive function/cognitive control	Academic achievement (reading, math)	Neurocognitive/indirect autonomic	Behavioral + cognitive testing	Strong evidence linking PA intervention to cognition and academic performance	Mechanistic measures indirect (no direct HRV/biomarkers)	Inferred
Jeon et al. (2017) [15]	Adolescents	Physical activity/exercise intensity	Interventional study	Memory performance	Not directly assessed	Neuroendocrine	Brain-derived neurotrophic factor (BDNF) levels	Demonstrates that exercise intensity modulates BDNF responses, supporting a neurobiological pathway linking physical activity to cognitive enhancement.	Limited assessment of academic outcomes and relatively short intervention duration	Direct
Davis et al. (2011) [25]	Overweight children (school-aged)	Structured physical activity intervention	Randomized controlled trial	Executive function	Academic achievement (mathematics and reading performance)	Neuroendocrine/brain-based	Neuroimaging (fMRI) and cognitive testing	Demonstrates that exercise-induced improvements in executive function are accompanied by changes in brain activation patterns	Sample limited to overweight children, reducing generalizability	Direct
Chaddock et al. (2010) [26]	Preadolescent children	Aerobic fitness/habitual physical activity	Cross-sectional study	Memory performance	Not directly assessed	Neuroendocrine/brain-based	Structural neuroimaging (hippocampal volume)	Links higher aerobic fitness to greater hippocampal volume and improved memory, supporting a structural brain mechanism	Cross-sectional design prevents causal inference	Direct

## Data Availability

No new data were created.

## Abbreviations

The following abbreviations are used in this manuscript:

BDNF	Brain-Derived Neurotrophic Factor
HRV	Heart Rate Variability
HPA	Hypothalamic–Pituitary–Adrenal
PRISMA-ScR	Preferred Reporting Items for Systematic Reviews and Meta-Analyses extension for Scoping Reviews
